# Radiological Findings of Malignant Tumors of External Auditory Canal: A Cross-Sectional Study Between Squamous Cell Carcinoma and Adenocarcinoma

**DOI:** 10.1097/MD.0000000000001452

**Published:** 2015-09-04

**Authors:** Shuang Xia, Shuo Yan, Mengjie Zhang, Yan Cheng, Jacinth Noel, Vincent Chong, Wen Shen

**Affiliations:** From the Departments of Radiology (SX, SY, MZ, JN, WS) and Otolaryngology, Tianjin First Central Hospital, Tianjin, China (YC) and Department of Diagnostic Radiology, National University Hospital, National University of Singapore, Singapore (VC).

## Abstract

The primary malignant tumors of external auditory canal (EAC) are rare. The purpose of this study is to compare the imaging features of growth and recurrence pattern between 2 most common carcinomas namely squamous cell carcinoma (SCC) and adenocarcinoma of the EAC.

This is a retrospective study involving 41 patients with primary EAC carcinomas of which 22 are SCC and 19 are adenocarcinoma. They were all scanned with high resolution computer tomography (HRCT) and magnetic resonance imaging. Follow-up clinical and imaging studies have also been collected and compared with a median follow-up time of 43 months (range 5–192 months). Necrosis was presented as hypodensity on computed tomography images, hyper-intense on T2WI and heterogeneous enhancement.

Eighteen patients were diagnosed to be in T1 and T2 stage, it was found that SCC involved both the cartilaginous part and the bony part of the EAC (11/12), whereas adenocarcinoma involved only the cartilaginous part (6/6) (*P* < 0.01). Twenty-three patients were diagnosed to be in T3 and T4 stage showed bony involvement and adjacent tissue involvement for both SCC and adenocarcinoma. Parapharyngeal space involvement is much more common in recurrent SCC (*P* = 0.02). Lymph node metastasis was seen in 6 out of 22 patients with SCC, while 5 out of 19 patients of adenocarcinoma had lung metastasis, even at early stage (1/6; 1/5). Necrosis is more likely to occur in the patients with SCC (9/10) than that of adenocarcinoma (3/13) (*P* = 0.02).

SCC and adenocarcinoma is seen to have different growth pattern at early stage but share similar patterns in the advanced stage. Lymph node metastasis is commonly seen in patients with SCC while adenocarcinoma shows lung metastasis even at early stage.

## INTRODUCTION

Primary malignant tumors of the external auditory canal (EAC) are rare and the incidence is <1 per million per year.^1,2^ Squamous cell carcinoma (SCC) is the most common primary malignant tumor and it accounts for 80% of tumors of EAC.^3,4^ Cystic adenocarcinoma is the second most common tumor which accounts for only 5%.^2,3,5–11^ Due to the rarity of these 2 entities, there are only a few studies comparing their differences in growth and recurrence pattern.^8,12,13^

The staging of malignant tumors of the EAC remains controversial.^1^ In 1990, Arriaga et al^5^ proposed a staging system based on computed tomography (CT) and pathology. This was followed by the addition of only minor revisions by Moody et al^3^ in 2000. Although surgical methods, selection of radiotherapy or chemotherapy are related to prognosis,^9,14,15^ studies have also shown that tumor extent, erosion of bone, and brain involvement to be directly related to survival rate.^8,12,13,16–20^ In comparison to SCC of EAC, adenocarcinoma has a higher risk of recurrence and distant metastasis. Dong et al^21^ reported that 7 patients with adenocarcinoma of EAC had pulmonary metastasis, 6 of them eventually succumbed to the disease. The prognosis of adenocarcinoma is related with the age of onset, duration of symptoms, perineural spread, and soft tissue involvement. Hence, early detection is an important prognostic factor in the treatment of malignant tumors of the EAC.

The symptoms of EAC tumors are often insidious and can be clinically misdiagnosed.^22^ Although otoscopy could easily visualize the lesion, imaging examination is necessary to map the tumor extent. A review of the literature revealed only a few papers on the imaging appearances of the tumors of EAC.^23–25^ It is also not clear if there are any differences in the imaging appearances of SCC and adenocarcinoma. Our hospital is a large tertiary health center for treating the patients with tumors of ear nose and throat and also our study collected some subtypes of adenocarcinoma (adenoid cystic carcinoma, ceruminous adenocarcinoma, and apocrine adenocarcinoma). So we collected almost the equally number of cases of SCC and adenocarcinoma and hypothesize that there are differences in the invasive and recurrent behavior of these 2 types of malignancies. The intent of this paper is to show the differences between SCC and adenocarcinoma of EAC in terms of their invasive and recurrent behavior as seen on high resolution computer tomography (HRCT) and magnetic resonance imaging (MRI).

## MATERIALS AND METHODS

### Patients

A total of 47 patients with pathologically confirmed malignant tumors of EAC from January 1998 to April 2014 in our hospital are retrospectively reviewed. Six cases were excluded from our study because 4 are rare tumors of the EAC (1 case of Rhabdomyo-sarcoma, 1 case of malignant melanoma, 1 case of metastatic low differentiated carcinoma, and 1 case of leukemia cell invasion) and 2 cases were lost to follow-up. Cases treated by radiotherapy before operation was not included in the study. This study is approved by our local Institutional Review Board of Tianjin First Center Hospital. A letter of patient consent to take part in the research was appended in the record.

Axial and coronal HRCT along with plain and contrast MRI data before operation or biopsy is available for all patients (except for 2 cases when only CT was done in 1998 and 2001). The patients’ age, sex, main symptoms and signs along with duration, the operation methods, and the location of lesions are summarized in Table 1 . Thirty-nine patients underwent operation in our hospital. Surgical planning was heavily influenced by CT and MRI appearances. Two patients had biopsy and no operation was performed. Initial T staging (listed in Table 1 ) is based on imaging, operation, and pathology (University of Pittsburgh tumor, nodes, metastasis staging system).^5^ Lesions limited to the EAC without and with limited bone erosion are staged T1 and T2. Tumors eroding the osseous EAC (full thickness) with limited (<0.5 cm) soft tissue involvement, or tumors involving the middle ear and/or mastoid are staged T3. Tumors with extensive bone and soft tissue involvement, such as cochlea, petrous apex, medial wall of the middle ear, carotid canal, jugular foramen, or dura are considered T4. Local complete resection (LCR) indicates that total tumor was resected but keeping the wall of EAC for tumor of stage1. Local external canal resection (LECR) indicates that total tumor was resected and combined with local involved EAC. Lateral temporal bone resection (LTBR) indicates that total tumor was resected and combined with lateral part of temporal bone. Partial parotid gland and soft tissue resection (PPGR) was performed if these tissue were involved. The detailed operation method for each case was summarized in Table 1 . The patients with T3 and T4 adenocarcinoma accepted radiotherapy in other hospital and the detailed dose is unavailable.

**TABLE 1:**
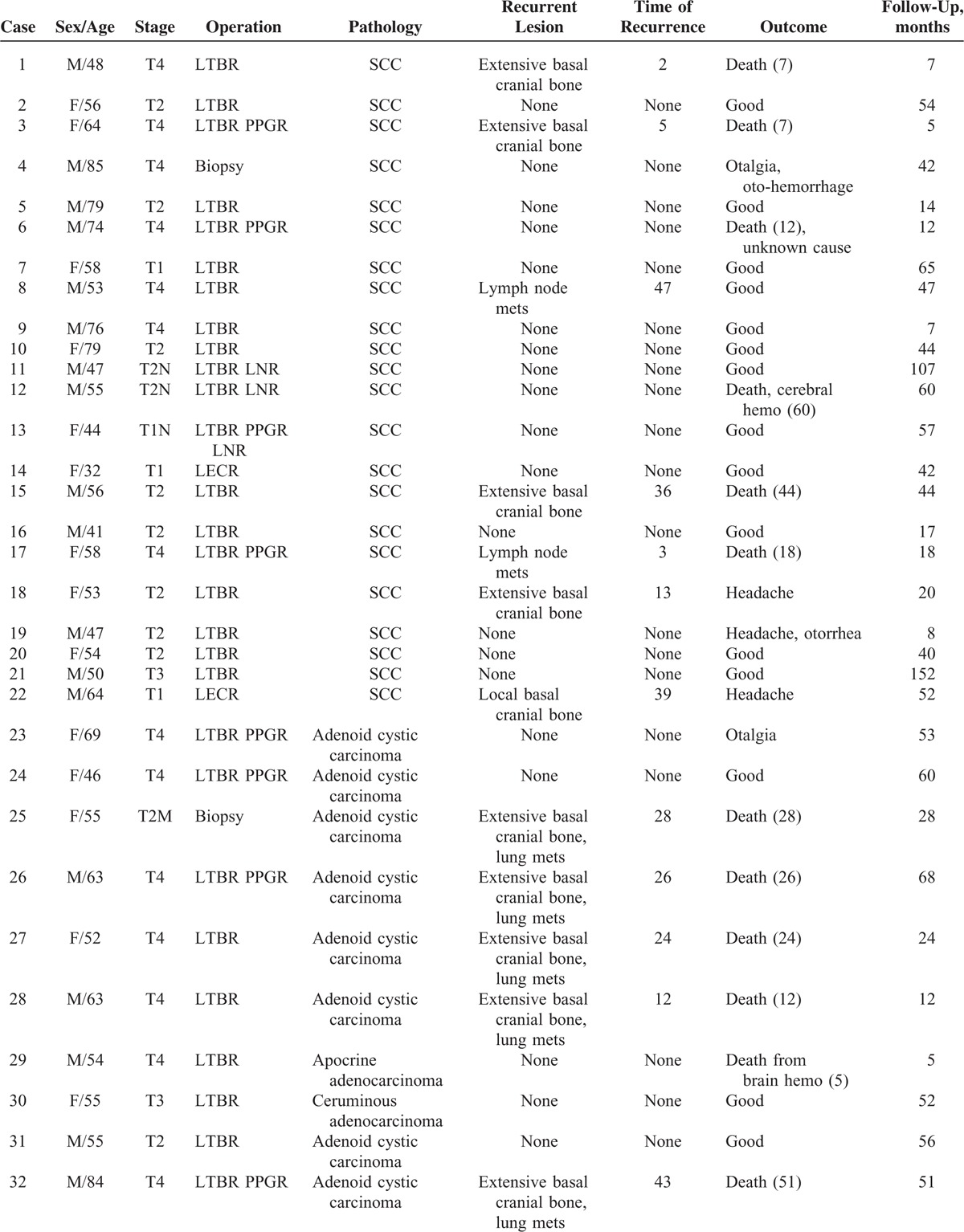
Clinical Characteristics of 41 Malignant Tumors of External Auditory Canal

**TABLE 1 (Continued):**
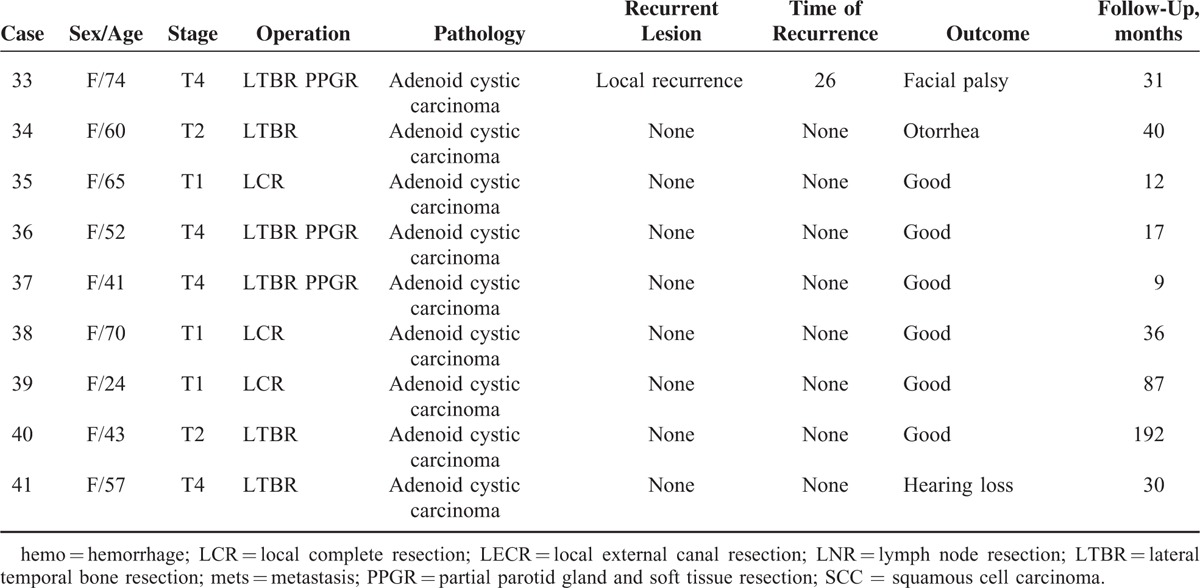
Clinical Characteristics of 41 Malignant Tumors of External Auditory Canal

The patients underwent regular follow-up until April 2014. Follow-up was done every year and general clinical symptoms and physical examinations were recorded. If the patients had symptoms at the time of follow-up, CT was performed. Patients with suspected recurrence on CT were usually further investigated with MRI. Median follow-up was 43 months (range, 5–192 months).

Finally, 41 cases were included in our study. There were 22 cases of SCC and 19 cases of adenocarcinoma (17 cases of adenoid cystic carcinoma, 1 case of ceruminous adenocarcinoma, and 1 case of apocrine adenocarcinoma). Among the 22 cases of SCC, 13 were males and 9 were females with mean age of 58.1 years (range, 32–85 years). Among the 19 cases of adenocarcinoma, 5 were males and 14 were females. The mean age at the time of diagnosis was 56.9 years (range, 24–84 years). Ten cases of SCC involved the left EAC and 12 cases the right ear. Eleven cases of adenocarcinoma were located in the left EAC and 8 in the right (Table 2).

**TABLE 2:**
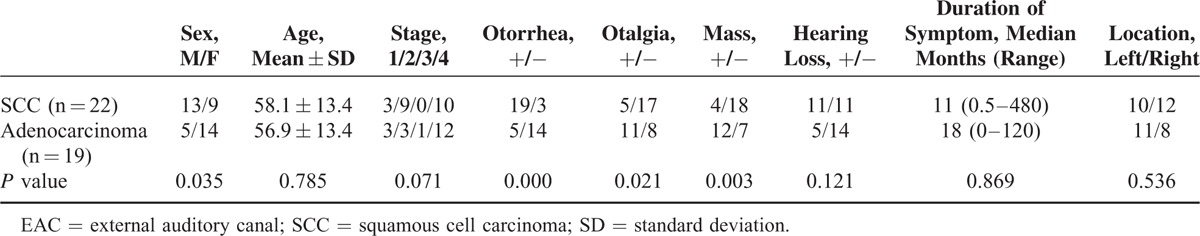
Comparison of Clinical Symptoms Between SCC and Adenocarcinoma of EAC

### HRCT and MRI Parameters

Axial HRCT performed (LightSpeed 16, GE Healthcare, Milwaukee, WI) with 120 kV and 140 mA, and the thickness 0.625 mm and 0 mm interval with bone algorithm. The scan range covers the mastoid apex and the skull base. Coronal images are reconstructed to check the superior and inferior walls of EAC.

MRI performed on 1.5T system (Eclipse, Marconi Medical Systems, Cleveland, OH) in 16 cases, and on 3T system (Trio Tim, Siemens AG, Erlangen, Germany) in 25 cases. The thickness is 3 mm and internal 0 mm. The coverage includes cranial base, brain, and neck to evaluate the area of the lesion. Axial and coronal spin-echo T1WI is acquired with the following parameters: TR 400 msec, TE 10 msec, matrix 256 × 256. Gradient echo T1WI is also employed to check the relationship between the lesion and adjacent vessels (TR 30 msec, TE 5.0 msec, matrix 256 × 256). Coronal and axial fat spin echo T2WI has been acquired with following parameters: TR 5307 msec, 900 msec, matrix 512 × 512, and thickness 3 mm.

Contrast MR scanning performed with intravenous injection of 0.1 mmol/kg contrast medium (Magnevist, Schering, Berlin, Germany) per kilogram of body weight in 35 patients.

### Imaging Interpretation

Bone destruction and soft tissue involvement and necrosis were evaluated independently by 2 experienced neuro-radiologists (10 and 16 years) who were unaware of the pathological results. In the case of disagreement, consensus to a more experienced neuro-radiologist (with an experience of 16 years) was reached and recorded. Location of bone destruction on CT included: anterior and posterior wall, superior and inferior wall, tempo-mandible joint (TMJ), squamous part of temporal bone, petrous and cranial basal bone, middle ear, mastoid, jugular fossa, and carotid canal. MRI evaluates the soft tissue involvement, including the middle ear, subcutaneous area, TMJ, parotid gland, dura, and parapharyngeal space. Necrosis was presented as hypodensity on CT images, hyper-intense on T2WI, and heterogeneous enhancement.

### Statistical Analysis

Image interpretations for inter-rater agreement were analyzed using kappa test. The differences in clinical and imaging characteristics were analyzed using independent Student *t* test. Fisher's exact test and Spearman Chi-squared test were used to compare clinical symptoms, growth and recurrent behavior and outcome between SCC and adenocarcinoma. A *P* value <0.05 were considered as statistically significant.

## RESULTS

### Clinical Data

No statistical difference was found between evaluations of location of bone destruction and soft tissue involvement by 2 independent radiologists (*P* > 0.05). The kappa value was 0.809 (*P* < 0.001). There is significant difference between gender for patients with SCC and adenocarcinoma (*P* = 0.04). Adenocarcinoma is much more commonly seen in females. No significant differences in age is noted at the initial time of diagnosis, along with the location of the tumor and duration of symptoms between SCC and adenocarcinoma (*P* = 0.79, 0.54, and 0.87, respectively). Patients presenting with otalgia and a mass is much more commonly seen in patients with adenocarcinoma (*P* = 0.02 and 0.01). However, SCC has significantly higher frequency of otorrhea compared with adenocarcinoma (19/3 vs 5/14, *P* < 0.01). Hearing loss is also associated with otorrhea and otalgia in both kinds of tumors (*P* = 0.12; Table 2). The medium duration of clinical symptoms is 42 months (range, 5–152 months) and 36 months (range, 5–192 months) for SCC and adenocarcinoma, respectively, which is statistically not significant (*P* = 0.80; Table 2). At the time of initial diagnosis, no differences are seen between the 2 types of tumors based on their tumor staging (*P* = 0.07).

### CT and MRI Findings of SCC and Correlation With T Stage

**FIGURE 1:**
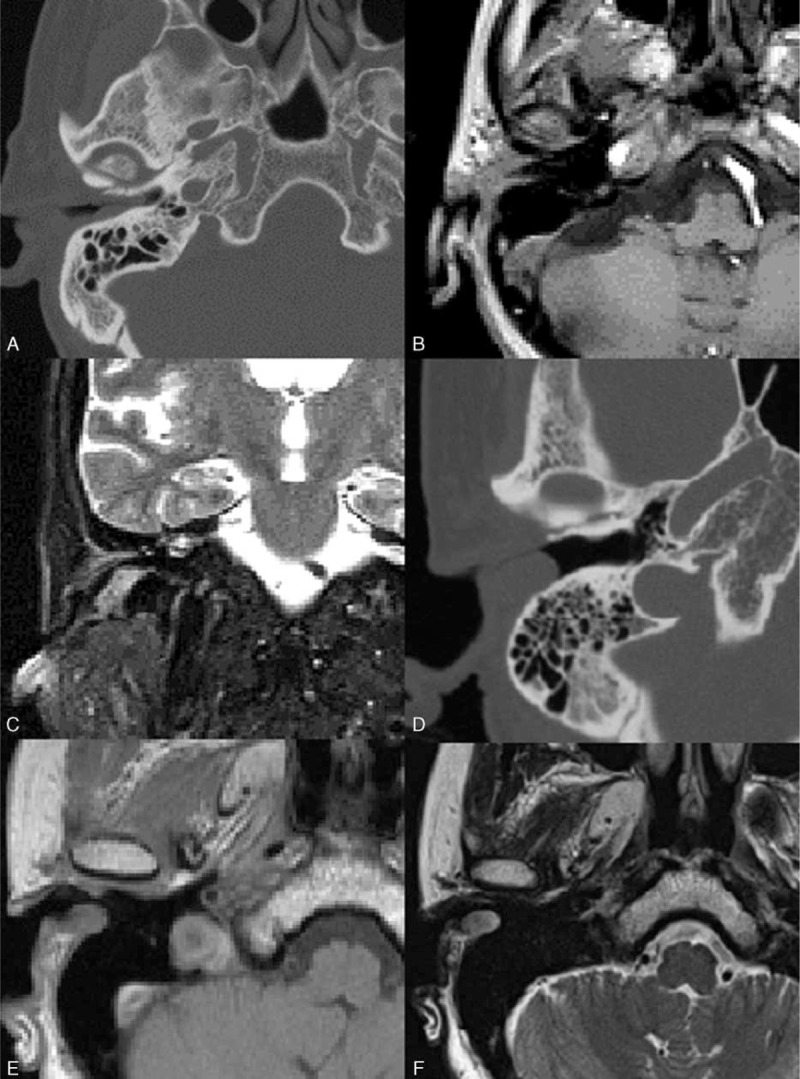
A 79-year-old man (case 5, SCC, stage 2) with complaints of otorrhea and otalgia for 12 months. HRCT and MRI were done (A–C). Axial HRCT revealed a soft tissue mass and stenosis of right EAC and (A). The lesion is iso-intense on T1WI (B) and hyper-intense on fat suppression T2WI (C). The lesion reaches the bony part of the EAC and involves the inferior wall of the EAC. A 65-year-old woman (case 35, cystic adenocarcinoma, stage 1) with complaints of otalgia and mass in her right ear for 1 month. HRCT and MRI were done (D–F). Axial HRCT (D) shows a focal mass locating at cartilage part of the EAC without bone changes. The lesion shows homogeneous signal intensity on both T1WI (E) and T2WI (F). EAC = external auditory canal; HRCT = high resolution computer tomography; MRI = magnetic resonance imaging; SCC = squamous cell carcinoma.

In 18 patients (with T1 and T2 tumors), the lesions are confined to the EAC with or without EAC wall erosion. SCC tends to involve both the cartilaginous and bony part of EAC (11/12) while in adenocarcinoma only the cartilaginous part is involved (6/6) (*P* < 0.01; Figure 1). No statistical differences is seen in the EAC wall involvement for SCC and adenocarcinoma (*P* = 0.57 for anterior wall of EAC, *P* = 0.56 for posterior wall of EAC, *P* = 0.56 for superior wall of EAC, and *P* = 0.56 for inferior wall of EAC). Both SCC and adenocarcinoma show isointensity on T1WI, slight hyper-intensity on T2WI (Figure 1), and homogenous contrast enhancement.

In 23 patients (T3 and T4 tumors), the wall of EAC is the most commonly involved structure. Wall of the EAC destruction shows no difference between SCC and adenocarcinoma (*P* = 0.46 for anterior wall of EAC, *P* = 0.54 for posterior wall of EAC, *P* = 0.47 for superior wall of EAC, and *P* = 0.61 for inferior wall of EAC). No statistical differences of the involved bone structure and soft tissue were found between 2 kinds of tumors (Table 3). The growth pattern in the advanced stage is similar for both tumors.

**TABLE 3:**
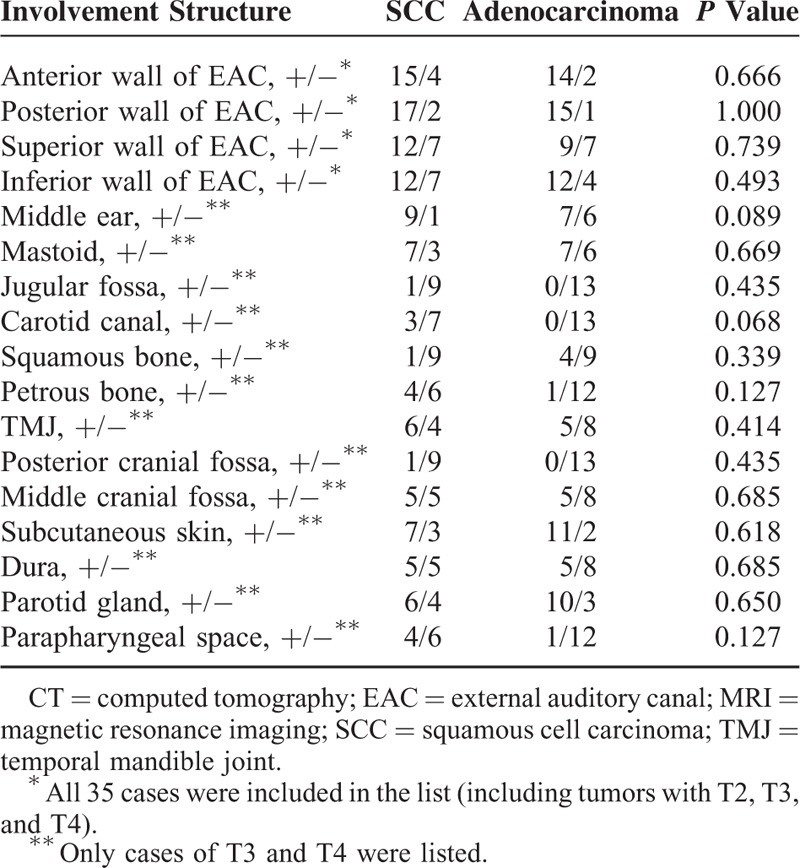
Comparison of Bone and Soft Tissue Involvement Between SCC and Adenocarcinoma of EAC on Both CT and MRI

**FIGURE 2:**
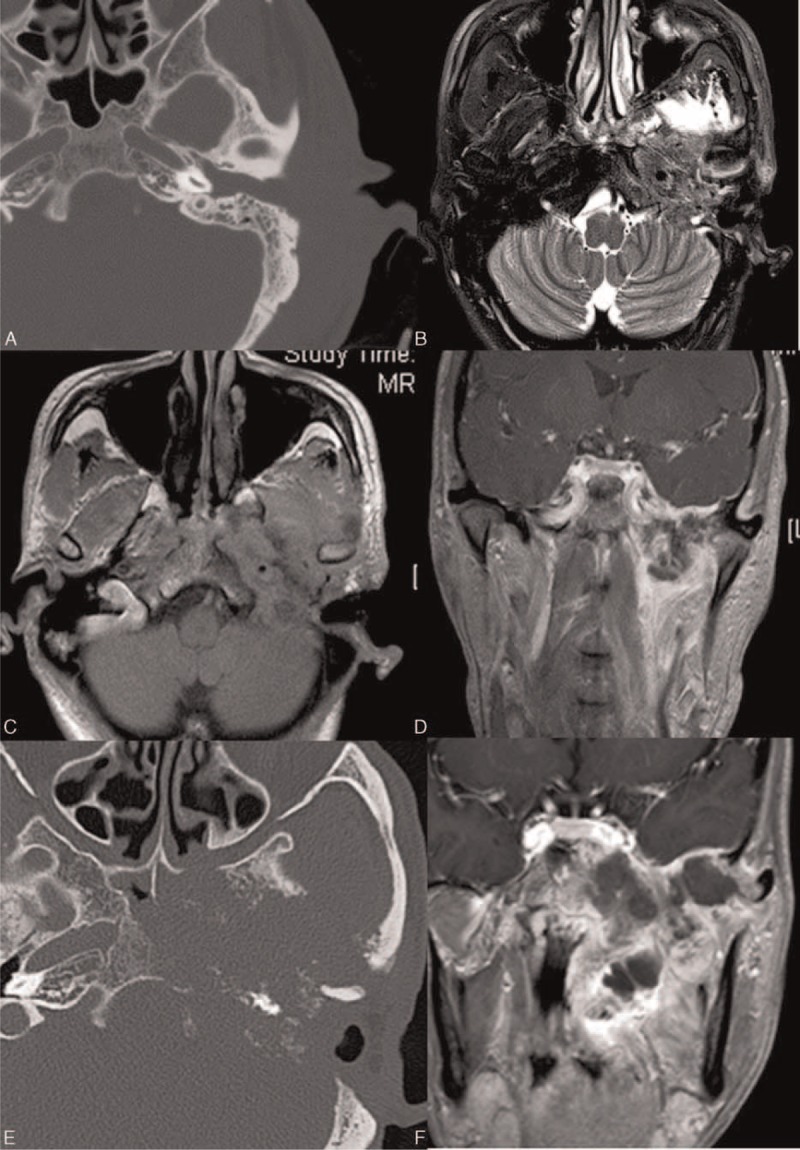
A 48-year-old man (case 1, SCC, stage 4) with complaints of otalgia and otorrhea of left ear for 1 month which worsened over last 5 days before presentation. Axial HRCT and MRI were done. He died 7 months later due to the recurrence. A large soft tissue mass is seen in the left EAC combined with anterior and posterior wall destruction (A). The lesion extensively involves the parapharyngeal space, subcutaneous area, encasing the sigmoid sinus, jugular fossa, and carotid artery without clear margin (B, C). Dura is also involved on contrast-enhanced MRI and the lesion shows heterogeneous enhancement (D). Lateral temporal bone resection was performed and pathologically confirmed to be SCC. Six months later, the patient complained of severe otalgia, fever and palsy of the cranial nerve III, IV, V IX, X, and XI. HRCT (E) shows extensive moth-eaten pattern of bone destruction including petrous bone, occipital clivus, sphenoid body, and carotid canal. Necrosis is noted on contrast enhancement MRI (F). EAC = external auditory canal; HRCT = high resolution computer tomography; MRI = magnetic resonance imaging; SCC = squamous cell carcinoma.

**FIGURE 3:**
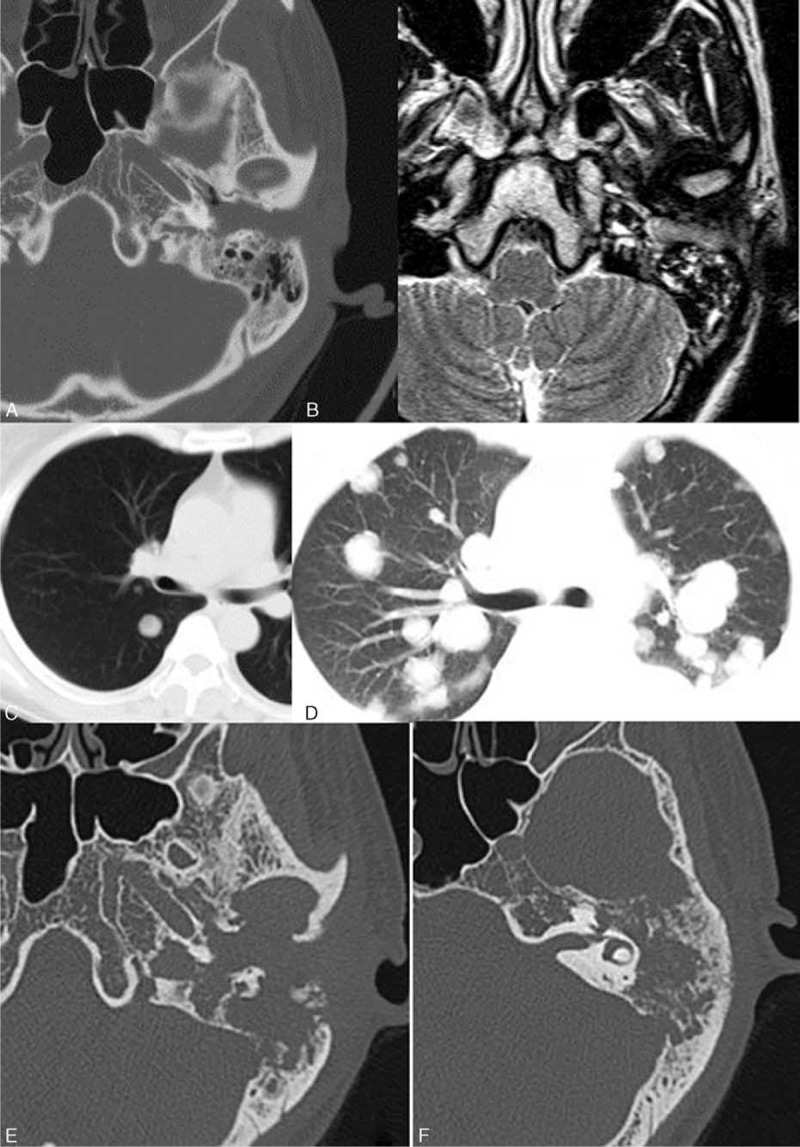
A 55-year-old female (case 25, cystic adenocarcinoma, stage 2) with complaints of mass in the left EAC for half a year with hearing loss for 4 months. HRCT and MRI are done. Axial HRCT reveals a soft tissue mass in the left EAC (A) with irregular destruction of the anterior and posterior wall of EAC. The lesion is iso-intense and locates in the left EAC as seen on T2WI (B). Multiple nodular metastases are seen on the lung CT (C). Operation was not done and only traditional medicine was given. Extensive metastases to the lungs (D) and temporal bone destruction are detected on the 28th month of follow-up (E). The patient died from lung metastases and local recurrence 28 months later. CT = computed tomography; EAC = external auditory canal; HRCT = high resolution computer tomography; MRI = magnetic resonance imaging.

The lesions in the T3 and T4 stages extensively involved the temporal subcutaneous area without any clear demarcation. Most of the lesions appeared iso-intense on T1WI and heterogeneously hyper-intense on T2WI for SCC and adenocarcinoma. Heterogeneous enhancement (due to necrosis) is seen in 9 of 10 cases of SCC and 3 of 13 cases of adenocarcinoma. Significant differences is seen on the contrast behavior pattern (homogenous and heterogeneous enhancement) between 2 kinds of tumors (*P* = 0.02; Figures 2 and 3).

### Comparison of Involved Structures in Local Recurrence and Clinical Outcome Between Patients With SCC and Adenocarcinoma

Local recurrence is seen in 7 patients with SCC (2 at stage 2 and 5 at stage 4) and 6 patients with adenocarcinoma (all at stage 4). Local recurrence in both SCC and adenocarcinoma is extensive (Table 4). Parapharyngeal space involvement is much more common in SCC (*P* = 0.02). Other structures such as jugular fossa, carotid canal, petrous bone, posterior and middle cranial fossa, subcutaneous soft tissue, parotid gland, TMJ, and dura were equally involved in the recurrent lesions for both kinds of tumors (Tables 4 and 5).

**TABLE 4:**
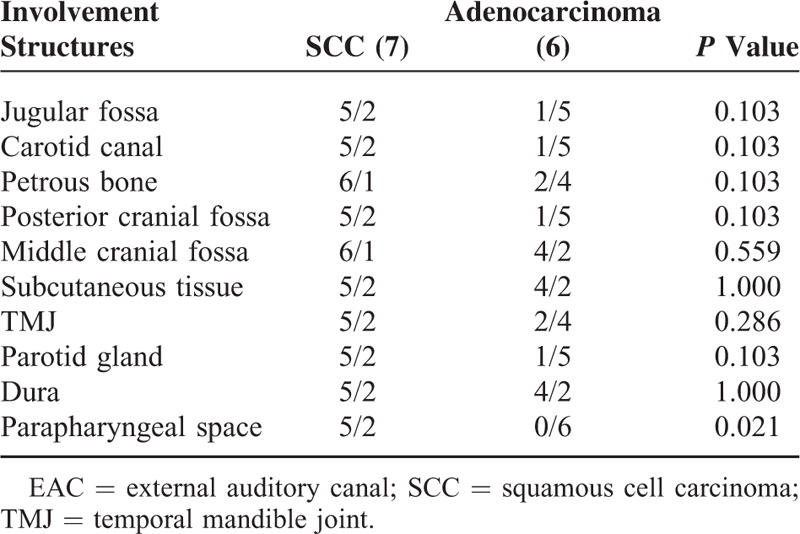
Comparison of Involvement Structure at Occurrence Between SCC and Adenocarcinoma of EAC

**TABLE 5:**
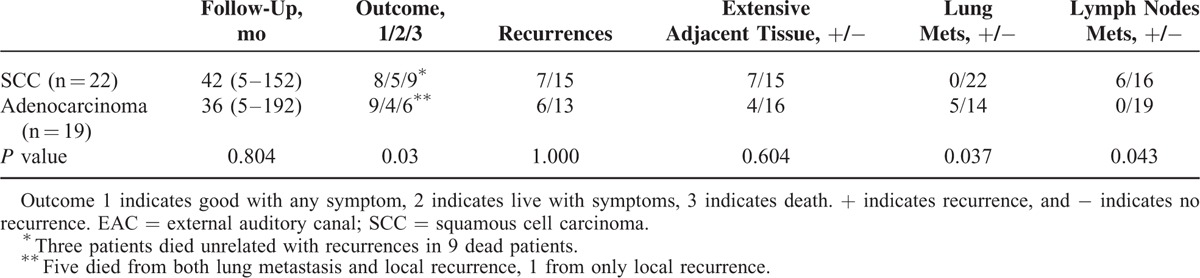
Comparison of Outcome Between SCC and Adenocarcinoma of EAC

Lymph node metastasis is seen in 6 cases of SCC and 2 cases in stage 2, 1 case in stage 1 but none in adenocarcinoma. In contrast to SCC, 5 patients with adenocarcinoma have lung metastasis and 1 case at stage 2 EAC adenocarcinoma (Figure 3).

The median follow-up time is 42 months (from 5 to 152 months) and 36 months (from 5 to 192 months) for SCC and adenocarcinoma, respectively. Nine patients with SCC died but 3 unrelated to tumor. Eight patients with SCC lived well after operation with good quality of life and 5 lived with symptoms. All 6 patients with adenocarcinoma died as a result of the tumor. Nine patients with adenocarcinoma had a good quality life after surgery while 4 patients lived with symptoms (Table 5).

## DISCUSSION

In this paper, the growth behavior and pattern of recurrence of SCC and adenocarcinoma has been comparatively studied based on their radiological findings. Adenocarcinoma has the tendency to grow on the superficial part of the EAC while SCC is more likely to grow along the entire length of the EAC and involving both the cartilaginous and bony part of the EAC at the early stage. These differences are very helpful for surgical planning. The operation method of LCR may be suitable for the adenocarcinoma of T1 stage but LECR or LTBR are more suitable for SCC of T1 stage. An accurate evaluation of the extent of malignant lesions of EAC is essential for treatment planning and prognosis.^26^ There are several proposed classification systems for staging malignancies of EAC.^3,5,18,27,28^ They have been used by many investigators.^3,8,20,29–36^ However, using a uniform staging system might ignore important behavioral differences between the malignancies of EAC resulting in less than optimal management.^33^

In the advanced stage, SCC is more likely to involve the deep structure extensively, such as parapharyngeal space. However, they both equally involve other structures, such as subcutaneous tissue, parotid gland, petrous bone along with and carotid artery wall. It is also noted that recurrent SCC and adenocarcinoma do not show distinct biological differences.

Lymph node metastasis is much more commonly seen in SCC even if the primary lesions are at an early stage. Nodal spread was seen in 6 cases with SCC but none in adenocarcinoma. Three patients showed nodal metastasis at initial diagnosis (1 T1 and 2 T2 tumors). A further 3 patients showed nodal metastasis on follow-up (all T4 tumors).

Adenocarcinoma is much more likely than SCC to metastasize to the lung and this feature can be seen at an early stage (1 patient had a T2 tumor). Lung metastasis was seen in 6 patients with adenocarcinoma. Our observation is similar to other reports documenting lung metastasis in patients with adenocarcinoma.^9,11,21^

Imaging plays an important role in delineating EAC tumor extent. Axial and coronal HRCT can demonstrate clearly destruction of the temporal bone.^23,24,37^ Both axial and coronal T1WI can show involvement of subcutaneous tissues, parotid gland, and parapharyngeal space. T2WI is sensitive to detect necrosis of the lesion, which shows heterogeneous hyperintensity. Contrast MRI is the best sequence for identifying dural or cerebral parenchymal invasion. Thickening of the dura and nodular contrast enhancement are signs of dural and cerebral invasion.^2,24,37^ In this study, we observed the tendency of SCC to undergo necrosis. Necrosis is usually seen in the central part of the lesion as areas of heterogeneous hyperintensity on T2WI and contrast enhancement on T1WI. Necrosis in adenocarcinoma is uncommon.

This study shows adenocarcinoma (compared to the patients with SCC), is more commonly seen in females (14/19). Otorrhea is the most common symptom in patients with SCC while otalgia and mass is the common presenting complaint in patients with adenocarcinoma. The results are similar to other reports.^1,2,11,21,38^

## CONCLUSIONS

SCC and adenocarcinoma have different growth behavior during early stage. In late stages, SCC is more likely to involve deep spaces such as the parapharyngeal space. The growth pattern, however, is similar for both types of tumors during recurrence. Lymph node metastasis is commonly seen in patients with SCC while lung metastasis is common in adenocarcinoma. Lymph nodes and lung metastasis could be seen in the early stage of the tumor. Necrosis is more likely to occur in the patients with SCC.

## LIMITATIONS

There are some limitations in the present study. First, the number of cases is still limited and more data should be collected to compare. Second, the subtype of adenocarcinoma should be divided in order to compare in more detail. Third, not all of the tumor were operated pending on the same criteria, such as the operation methods for tumors of T1 stage included LCR, LECR, and LTBR. Fourth, the dose of radiation therapy for tumors of T3 and 4 is unclear. More detail data of patients will be collected to make further analysis.

## References

[1] Ouaz K Robier A Lescanne E Cancer of the external auditory canal. *Eur Ann Otorhinolaryngol Head Neck Dis* 2013; 130:175–182. 23845289 10.1016/j.anorl.2012.08.003

[2] Kuhel WI Hume CR Selesnick SH Cancer of the external auditory canal and temporal bone. *Otolaryngol Clin North Am* 1996; 29:827–852. 8893219

[3] Moody SA Hirsch BE Myers EN Squamous cell carcinoma of the external auditory canal: an evaluation of a staging system. *Am J Otol* 2000; 21:582–588. 10912706

[4] Visnyei K Gill R Azizi E Squamous cell carcinoma of the external auditory canal: a case report and review of the literature. *Oncol Lett* 2013; 5:1587–1590. 23761823 10.3892/ol.2013.1241 PMC3678866

[5] Arriaga M Curtin H Takahashi H Staging proposal for external auditory meatus carcinoma based on preoperative clinical examination and computed tomography findings. *Ann Otol Rhinol Laryngol* 1990; 99 (9 Pt 1):714–721. 2396807 10.1177/000348949009900909

[6] Shih L Crabtree JA Carcinoma of the external auditory canal: an update. *Laryngoscope* 1990; 100:1215–1218. 2233086 10.1288/00005537-199011000-00016

[7] Testa JR Fukuda Y Kowalski LP Prognostic factors in carcinoma of the external auditory canal. *Arch Otolaryngol Head Neck Surg* 1997; 123:720–724. 9236591 10.1001/archotol.1997.01900070064010

[8] Nyrop M Grontved A Cancer of the external auditory canal. *Arch Otolaryngol Head Neck Surg* 2002; 128:834–837. 12117346 10.1001/archotol.128.7.834

[9] Gu FM Chi FL Dai CF Surgical outcomes of 43 cases with adenoid cystic carcinoma of the external auditory canal. *Am J Otolaryngol* 2013; 34:394–398. 23453117 10.1016/j.amjoto.2013.01.018

[10] Kagotani A Ishida M Yoshida K Adenoid cystic carcinoma of the external auditory canal successfully diagnosed by fine-needle aspiration. *Diagn Cytopathol* 2014; 42:102–104. 22865786 10.1002/dc.22909

[11] Liu SC Kang BH Nieh S Adenoid cystic carcinoma of the external auditory canal. *J Chin Med Assoc* 2012; 75:296–300. 22721626 10.1016/j.jcma.2012.04.007

[12] Moore MG Deschler DG McKenna MJ Management outcomes following lateral temporal bone resection for ear and temporal bone malignancies. *Otolaryngol Head Neck Surg* 2007; 137:893–898. 18036417 10.1016/j.otohns.2007.09.010

[13] Pensak ML Gleich LL Gluckman JL Temporal bone carcinoma: contemporary perspectives in the skull base surgical era. *Laryngoscope* 1996; 106:1234–1237. 8849792 10.1097/00005537-199610000-00012

[14] Takenaka Y Cho H Nakahara S Chemoradiation therapy for squamous cell carcinoma of the external auditory canal: a meta-analysis. *Head Neck* 2015; 37:1073–1080. 24692266 10.1002/hed.23698

[15] Spielmann PM McKean S White RD Surgical management of external auditory canal lesions. *J Laryngol Otol* 2013; 127:246–251. 23351401 10.1017/S0022215112003155

[16] Moffat DA Wagstaff SA Hardy DG The outcome of radical surgery and postoperative radiotherapy for squamous carcinoma of the temporal bone. *Laryngoscope* 2005; 115:341–347. 15689763 10.1097/01.mlg.0000154744.71184.c7

[17] Zhang B Tu G Xu G Squamous cell carcinoma of temporal bone: reported on 33 patients. *Head Neck* 1999; 21:461–466. 10402528 10.1002/(sici)1097-0347(199908)21:5<461::aid-hed13>3.0.co;2-l

[18] Kunst H Lavieille JP Marres H Squamous cell carcinoma of the temporal bone: results and management. *Otol Neurotol* 2008; 29:549–552. 18520589 10.1097/MAO.0b013e31816c7c71

[19] Gillespie MB Francis HW Chee N Squamous cell carcinoma of the temporal bone: a radiographic-pathologic correlation. *Arch Otolaryngol Head Neck Surg* 2001; 127:803–807. 11448354

[20] Nakagawa T Kumamoto Y Natori Y Squamous cell carcinoma of the external auditory canal and middle ear: an operation combined with preoperative chemoradiotherapy and a free surgical margin. *Otol Neurotol* 2006; 27:242–248.discussion 249. 16436996 10.1097/01.mao.0000190463.88873.3d

[21] Dong F Gidley PW Ho T Adenoid cystic carcinoma of the external auditory canal. *Laryngoscope* 2008; 118:1591–1596. 18677277 10.1097/MLG.0b013e31817c42a8

[22] Zhang T Dai C Wang Z The misdiagnosis of external auditory canal carcinoma. *Eur Arch Otorhinolaryngol* 2013; 270:1607–1613. 22926989 10.1007/s00405-012-2159-4

[23] Arriaga M Curtin HD Takahashi H The role of preoperative CT scans in staging external auditory meatus carcinoma: radiologic-pathologic correlation study. *Otolaryngol Head Neck Surg* 1991; 105:6–11. 1909009 10.1177/019459989110500102

[24] Leonetti JP Smith PG Kletzker GR Invasion patterns of advanced temporal bone malignancies. *Am J Otol* 1996; 17:438–442. 8817022

[25] Ong CK Pua U Chong VF Imaging of carcinoma of the external auditory canal: a pictorial essay. *Cancer Imaging* 2008; 8:191–198. 18940738 10.1102/1470-7330.2008.0031 PMC2590879

[26] Gandhi AK Roy S Biswas A Treatment of squamous cell carcinoma of external auditory canal: a tertiary cancer centre experience. *Auris Nasus Larynx* 2015; Epub ahead of print. 10.1016/j.anl.2015.06.005 26165629

[27] Stell PM McCormick MS Carcinoma of the external auditory meatus and middle ear. Prognostic factors and a suggested staging system. *J Laryngol Otol* 1985; 99:847–850. 4045304 10.1017/s0022215100097796

[28] Manolidis S Pappas D Jr Von Doersten P Temporal bone and lateral skull base malignancy: experience and results with 81 patients. *Am J Otol* 1998; 19 Suppl:S1–S15. 9827809

[29] Austin JR Stewart KL Fawzi N Squamous cell carcinoma of the external auditory canal. Therapeutic prognosis based on a proposed staging system. *Arch Otolaryngol Head Neck Surg* 1994; 120:1228–1232. 7917206 10.1001/archotol.1994.01880350036007

[30] Pfreundner L Schwager K Willner J Carcinoma of the external auditory canal and middle ear. *Int J Radiat Oncol Biol Phys* 1999; 44:777–788. 10386634 10.1016/s0360-3016(98)00531-8

[31] Pemberton LS Swindell R Sykes AJ Primary radical radiotherapy for squamous cell carcinoma of the middle ear and external auditory cana—an historical series. *Clin Oncol (R Coll Radiol)* 2006; 18:390–394. 16817330 10.1016/j.clon.2006.03.001

[32] Hashi N Shirato H Omatsu T The role of radiotherapy in treating squamous cell carcinoma of the external auditory canal, especially in early stages of disease. *Radiother Oncol* 2000; 56:221–225. 10927141 10.1016/s0167-8140(00)00220-6

[33] Lim LH Goh YH Chan YM Malignancy of the temporal bone and external auditory canal. *Otolaryngol Head Neck Surg* 2000; 122:882–886. 10828803 10.1016/S0194-59980070018-0

[34] Birzgalis AR Keith AO Farrington WT Radiotherapy in the treatment of middle ear and mastoid carcinoma. *Clin Otolaryngol Allied Sci* 1992; 17:113–116. 1375135 10.1111/j.1365-2273.1992.tb01055.x

[35] Yin M Ishikawa K Honda K Analysis of 95 cases of squamous cell carcinoma of the external and middle ear. *Auris Nasus Larynx* 2006; 33:251–257. 16431060 10.1016/j.anl.2005.11.012

[36] Gabriele P Magnano M Albera R Carcinoma of the external auditory meatus and middle ear. Results of the treatment of 28 cases. *Tumori* 1994; 80:40–43. 8191597 10.1177/030089169408000108

[37] Gidley PW Managing malignancies of the external auditory canal. *Expert Rev Anticancer Ther* 2009; 9:1277–1282. 19761431 10.1586/era.09.93

[38] Bibas AG Ward V Gleeson MJ Squamous cell carcinoma of the temporal bone. *J Laryngol Otol* 2008; 122:1156–1161. 18177533 10.1017/S0022215107001338

